# Druggable proteins of *Alistipes* reveal promising antimicrobial targets against chronic intestinal inflammation

**DOI:** 10.3389/fphar.2025.1555062

**Published:** 2025-04-24

**Authors:** Saman Sohail, Ayesha Wisal, Sobia Rana, Rao Muhammad Abid Khan, Asad Ullah, Farooq-Ahmad Khan, Muhammad Irfan, Muhammad Imran, Paulo V. S. D. Carvalho, Renato Rozental, Farzana Shaheen, Syed Shah Hassan

**Affiliations:** ^1^ Department of Chemistry, Islamia College Peshawar, Peshawar, Pakistan; ^2^ Dr. Panjwani Center for Molecular Medicine and Drug Research, International Center for Chemical and Biological Sciences (PCMD-ICCBS), University of Karachi, Karachi, Pakistan; ^3^ Department of Clinical Microbiology, Sindh Institute of Urology and Transplantation (SIUT), Karachi, Pakistan; ^4^ TWC, HEJ-ICCBS, University of Karachi, Karachi, Pakistan; ^5^ Research Center for Advanced Materials Science (RCAMS), Department of Chemistry, Faculty of Science, King Khalid University, Abha Aseer, Saudi Arabia; ^6^ Centre for Technological Development in Health (CDTS), Oswaldo Cruz Foundation (Fiocruz), Rio de Janeiro, Brazil

**Keywords:** *Alistipes* genera, subtractive genomics, druggable targets, TCM/ZINC inhibitors, pharmacokinetics, thermodynamics evaluation

## Abstract

**Introduction:**

The genus *Alistipes* consists of anaerobic, Gram-negative bacteria with 13 species that colonize the entire gastrointestinal tract and are a serious health concern. They contribute to gut dysbiosis, intestinal inflammation, colorectal cancer, and depression.

**Methods:**

To explore potential therapeutic targets and inhibitors, we filtered the core genome of *Alistipes* strains through subtractive genomics for non-host homology, gene essentiality, PPI, KEGG pathways, virulence, cellular localization, and druggability. The potential targets were docked against two drug-like libraries (ZINC, n = 11,993) and TCM (n = 36,043). ADMET profiling for best hits and MD simulation for apo/complex structures were performed, followed by physicochemical and pharmacokinetic evaluation and complex stabilities.

**Results and Discussion:**

A set of 39 potential proteins was drastically reduced to only two targets after sequential data mining. The 3D structures of the selected targets (*LpxA* and *KdsB*) revealed good druggability scores. The top hits (ZINC85530940, ZINC05161112, ZINC95911713, and ZINC05566415) for both targets showed maximum H-bond interactions. The RMSD and RMSF values exhibited compactness with minimum fluctuation in ligand-bound complexes. The β-factor of ZINC05161112 at 327th residue and 352nd residue exhibited higher thermal instability, consistent with the RMSF results. The globularity of the complexes and apo structures remained consistent, whereas the *LpxA* complexes exhibited lower solvent-accessible surface area. For the *KdsB*, the surface area for ZINC5566415 increased significantly, with a steep decrease for ZINC95911713, establishing rather stable protein-ligand complexes. The results highlight the importance of identifying novel inhibitors and therapeutic targets. They are crucial for establishing better treatment regimes for human health and to aid in controlling the pathogenicity of *Alistipes* species.

## 1 Introduction

Humans acquire microbiota at birth, and during their life span, their gut is colonized by trillions of microorganisms, primarily bacteria. A broad array of bacterial phyla has been revealed using 16 S rRNA gene sequencing, with *Firmicutes* and *Bacteroidetes* being the most abundant (Goodrich et al., 2017). The human gut microbiota is important for immune response regulation, pathogen defense, digestive assistance, neurologic signaling, and vascularization (Lynch and Pedersen, 2016). An imbalance, or dysbiosis, in the gut microbiota has been linked to various disorders, including cardiovascular diseases (Halfvarson et al., 2017), inflammatory bowel disease (Yoshida et al., 2018), cancer (Vivarelli et al., 2019), and neurological disorders (Petra et al., 2015). Researchers have investigated various microbial genera and species within the microbiome to determine their role and identify specific bacterial species that influence disease progression and treatment response.

Among the diverse gut microbial populations, the genus *Alistipes* has gained attention due to its dual role in gut homeostasis and disease progression. This relatively new genus of bacteria from the *Bacteroidetes* phylum represents a group of Gram-negative, rod-shaped, anaerobic, and non-spore-forming bacteria. It has 13 species as of April 2020, according to the taxonomy database at the National Center for Biotechnology Information (NCBI) (Basson et al., 2020). While *Alistipes* species are primarily commensals in the gastrointestinal tract (GIT), their presence has been reported in appendicular, abdominal, perirectal, and brain abscesses, as well as in the bloodstream, urine, and peritoneal fluid, suggesting opportunistic pathogenicity (Shkoporov et al., 2015).

*Alistipes* species have also been implicated in gut dysbiosis and inflammatory disorders. For example, *Alistipes putredinis* has been identified in patients with appendicitis and abdominal abscesses, while *Alistipes onderdonkii* and *Alistipes shahii* were isolated from appendix tissue and urine samples (Rautio et al., 2003; Song et al., 2006). Several new *Alistipes* species have been identified in human fecal samples, including *Alistipes communis*, *Alistipes dispar*, *Alistipes megaguti*, and *Alistipes provencensis* (Bellali et al., 2019). The metabolic adaptability of *Alistipes*, including unique lipid biosynthesis pathways such as sphingolipid and sulfonolipid production, further distinguishes this genus from other gut microbes (Geiger et al., 2010).

Chronic intestinal inflammation, including inflammatory bowel diseases (IBD), is commonly treated with antibiotics, immunosuppressants, and biologic therapies. Antibiotics like metronidazole, ciprofloxacin, and rifaximin help regulate gut microbiota but face increasing resistance in gut bacteria, including *Alistipes,* which challenges their long-term effectiveness. Immunosuppressants (azathioprine, methotrexate, corticosteroids) control inflammation but do not address gut dysbiosis, while biologics (infliximab, adalimumab, vedolizumab) target immune pathways without eliminating opportunistic pathogens that may contribute to disease recurrence (Parker et al., 2020; Zhang et al., 2024).

Recent studies have highlighted the antibiotic resistance profiles of *Alistipes* species, demonstrating resistance to vancomycin, kanamycin, colistin, clindamycin, cefoxitin, and tetracycline, among others​. Their metabolic adaptability and resistance mechanisms make them an emerging concern in antimicrobial research. Despite their clinical relevance, limited research has focused on identifying potential drug targets for therapeutic intervention (Rautio et al., 2003; Mishra et al., 2012; Lagier et al., 2012; Pfleiderer et al., 2014; Jousimies-Somer, 2002).

The primary phase in the vaccine, drug, and diagnostic biomarker development process is the identification of targets of interest. For that purpose, an *in silico* subtractive proteomic approach is commonly used (Wisal et al., 2024; Hassan et al., 2018; Irfan et al., 2023; Basharat et al., 2022; Kalhor et al., 2023; Alqurashi et al., 2024). Many advantages of such approaches include cost-effectiveness, reduced time and labor, reproducibility, and robustness to yield broad-spectrum therapeutic candidates (Barh et al., 2010a; Barh et al., 2010b). This study employs *in silico* subtractive genomics to identify potential druggable targets in Alistipes species. Using core-genome analysis, essential gene filtering, protein-protein interaction (PPI) analysis, and druggability assessments, we aim to pinpoint novel antibacterial targets. Furthermore, molecular docking, ADMET profiling, and molecular dynamics simulations are utilized to evaluate promising inhibitors, ensuring their stability, pharmacokinetics, and binding affinity. This study provides a computational framework for developing targeted therapies against *Alistipes*-associated gut dysbiosis while minimizing disruption to the broader gut microbiome.

## 2 Approaches and methodologies

### 2.1 Strains selection and identification of core genome

In the present work, 9 *Alistipes* strains with complete genomes were included for the core-genome analysis. We selected complete genomes to ensure comprehensive core-genome and accurate data analysis. This allows us to capture the full genetic information, ensuring no data is missed and enhancing the reliability of our findings for a thorough investigation of unique, essential, and non-homologous proteins across *Alistipes* strains. For this study, nine *Alistipes* strains with complete genomes were randomly chosen, and the DNA, protein, and general genome statistics were acquired from the National Center for Biotechnology Information (NCBI), a public database freely available to the scientific community (https://www.ncbi.nlm.nih.gov/). This database contains manually curated protein sequences under the UniProtKB/Swiss-Prot and automatically annotated protein sequences under the UniProtKB/TrEMBL (Boutet et al., 2007). After the retrieval of complete genomes, the core genome was discovered through the pangenome approach in the PATRIC web server (Wattam et al., 2014). For the identification of the core genome, the PATRIC software provides a robust comparative analysis environment, allowing us to perform detailed comparisons of gene content, sequence variations, and functional annotations. The selection criteria in PATRIC are as follows: one strain is randomly chosen as the reference strain (*Alistipes finegoldii*), and the remaining strains are compared to it using the modified parameters, from which the core genes (shared by all strains) are filtered. The PATRIC employs the Basic Local Alignment Search Tool algorithm for protein sequences (BLASTp) with the standard scoring matrix BLOSUM62 and the following cutoff values: *e = 1 × 10* ^*−5*^, % *coverage ≥ 90%,* and *% identity ≥ 95%* (Mahram and Herbordt, 2015; Eddy, 2004).

### 2.2 Identification of essential and non-homologous genes

The Database of Essential Genes (DEG) server was used to eliminate the redundant proteins (Luo et al., 2021). The identified proteins were compared to Prokaryotes, Eukaryotes, and Archaea, using default settings such as *e-value* = *0.0001, bit score = 100,* and % *identity 35.* Only proteins that did not show a significant match to any of the Prokaryotes, Eukaryotes, and Archaea protein datasets were considered for inclusion (Luo et al., 2021). For homologous sequences exclusion, pathogen-specific and non-host homolog drug targets were filtered by subjecting the resultant sequences to the BLASTp (http://blast.ncbi.nlm.nih.gov/Blast.cgi) against human proteome (TaxID: 9,606) (*e-value*: 10^-4^), a step to avoid any cross-reactivity in a possible host (Kerfeld and Scott, 2011).

### 2.3 Cellular localization and PPI prediction

The subcellular localization of the unique, essential, and non-homologs was predicted using the CELLO2GO (v2.5) (http://cello.life.nctu.edu.tw/cello2go/) (Yu et al., 2014) and the PSORTb (v3.0.2) (http://www.psortb.org/psortb/) (Yu et al., 2010). The protein sequences were submitted in FASTA format with the organism type set to bacteria and Gram stain set to negative. For bacteria, protein subcellular localization prediction is the accurate tool, and it applies Support Vector Machines (SVMs) that assign a possible localization site to a protein. Furthermore, it assigns the five subcellular locations, i.e., Periplasm, extracellular, cytoplasm, inner membrane, and outer membrane, to Gram-negative bacteria. Parallel to the PSORTb, the CELLO2GO takes the functionality of the SVMs at two levels. The initial classification of a protein subcellular location is performed based on sequence-derived molecular descriptors, followed by a final decision centered on the probability of the subcellular location. Subcellular localization analysis was performed to refine target selection by identifying proteins based on their accessibility and functional relevance. We prioritized cytoplasmic proteins for further analysis, including protein-protein interaction (PPI) mapping and target selection. Cytoplasmic proteins were selected due to their essential roles in metabolic and regulatory pathways, making them viable candidates for small-molecule inhibitors. This approach ensured the selection of high-confidence therapeutic targets that are both functionally significant and pharmacologically accessible (Yu et al., 2004).

Protein-protein interaction (PPI) was performed using the STRING (Search Tool for the Retrieval of Interacting Genes/Proteins) database (v11.5) (https://string-db.org/), applying a minimum interaction confidence score of 0.7 (high confidence) to ensure reliable functional associations (Szklarczyk et al., 2019). STRING is a protein-protein interaction platform that contains both known and anticipated interactions. The interactions arise from computational prediction, information transfer between species, and interactions gathered from other (primary) databases, and they comprise both direct (physical) and indirect (functional) relationships. Only interactions supported by experimental evidence, database annotations, and computational predictions with a combined score above 0.7 were considered for further analysis. Additionally, nodes with fewer than three interactions were excluded to refine the interaction network and focus on biologically significant targets. This filtering approach ensured the selection of highly connected and functionally relevant proteins for downstream analysis (Szklarczyk et al., 2021).

### 2.4 Drug target prioritization for target selection

Several variables, including molecular function, molecular weight, pathway analysis, cellular localization, and virulence, were taken into consideration when determining prospective drug targets (Agüero et al., 2008). The ProtParam tool2 calculates the molecular weight (MW) (Gasteiger et al., 2005) for targets, and an MW ≤ 100 kDa is regarded as an ideal (Hossain et al., 2016; Mondal et al., 2015). In bacteria, any biological function of proteins is defined in the context of their location and function, where inhibition of such function requires the downregulation of molecular interaction partners due to the promiscuous nature of a target protein, which might impact their activity. On the other hand, for the physiological function of hypothetical and novel proteins, understanding protein function and virulence factors is often critical (Scott et al., 2005).

The potentially druggable proteins brought forth by the genomic screening carried information about their subcellular location and the metabolic pathways involvement, the UniProtKB was used to gather information regarding the function of the protein, catalytic requirements for enzymatic activity, and active isoforms (dependency on cofactors, subunit’s structure, and associated post-translational modifications) (Consortium, 2015). The VFDB tool (Virulence Factor Database) was employed to determine the virulence of target proteins (Chen et al., 2005a), while the MHOLline biological workflow (http://www.mholline2.lncc.br) was employed to group the proteins based on template similarity scores to identify the top drug candidates for 3D modeling (Hassan et al., 2014). All these steps allowed the choice of the current targets for CADD analysis (computer-aided drug design).

### 2.5 KEGG metabolic pathway analyses

Mining of potential drug targets depends highly on the categorization of proteins involved in pathogen-specific metabolic pathways. The KEGG Automatic Annotation Server (KAAS) was utilized to screen out the essential proteins for metabolic pathway analysis (Moriya et al., 2007). Kyoto Encyclopedia of Gene and Genome (KEGG) pathway database then further maps out proteins involved in host-specific pathways by comparative analysis of host and pathogen metabolic pathways (Kanehisa and Goto, 2002). The output files by the KAAS server comprised information such as the enzyme names, the Enzyme Commission (EC) numbers, alternative pathways, the KEGG Orthology (KO) list assignment, and the metabolic pathways. The obtained dataset symbolizes the non-homologous proteins involved in crucial pathways of *A. finegoldii* (Kanehisa et al., 2017; Kanehisa et al., 2017). The anticipated pathogen pathways were manually compared to human pathways, unique and common, where unique were and exclusive to the bacterium and, hence, the focus of the current work, where common pathways were those present in both the bacterium and the host.

In addition to the KEGG, the MetaCyc or BioCyc tools also provide comprehensive databases and precise and detailed annotations and have been used in some of our previous works (Shah Hassan et al., 2012; Pereira et al., 2013). However, in this work, we selected the KAAS due to its ability to map the KEGG pathways specific to bacterial species, which aligns with our focus on pathogen-specific pathways. It offers a comprehensive database and precise KO (KEGG Orthology) assignments, integrating data from the KEGG GENES database and providing reliable and detailed annotations. This approach provides a more consistent and comprehensive view compared to other tools like the MetaCyc/BioCyc. Furthermore, the final targets were blasted against the gut microbiota (a manually assembled fasta file containing 52,618 genes) to avoid impact on the gut microbiota using the following parameters: *e-value = 10, bit score = 200,* and *identity = 35%*.

### 2.6 3D structure modeling and energy minimization

The three-dimensional structures of the potential drug targets, *LpxA* and *KdsB* (locus IDs: Alfi_0084, Alfi_2459), were not available in the RCSB-PBD database (https://www.rcsb.org). Therefore, we followed the comparative homology modeling to predict the 3D structures using the respective amino acids or nucleotide sequences from the reference genome of *A. finegoldii.* The selected sequences were then subjected to a BLASTp search against the PDB database for the selection of a suitable template with the best sequence identity and coverage. Templates with sequence identity and query coverage of ≥90% were selected for structure modeling.

To identify the best therapeutic candidates, protein sequences were sorted based on their similarities to the templates using the MHOLline (v2.0) biological workflow (http://www.mholline2.lncc.br) as adapted from Hassan et al. (2014). This workflow performed quality-based sequence sorting, and the highest quality sequences were subjected to the SWISS-MODEL server (Arnold et al., 2006). The 3D structure models for the filtered protein targets were then generated and visualized using the molecular graphics tool PyMOL (https://pymol.org/2/). To check the reliability of the generated model, a validation step is crucial; hence, all models were evaluated using PDBSum (Laskowski, 2001), ERRAT value (Dym et al., 2012), Verify3D (Eisenberg et al., 1997), and ProSA (Wiederstein and Sippl, 2007). All these measurements were used in the selection of the best 3D model.

The selected models (*LpxA* and *KdsB*) were subjected to energy minimization to improve their qualities for further use in docking, etc., studies. A powerful visualization tool, UCSF Chimera, was used to analyze the structures (Pettersen et al., 2004) for minimized energy. Gasteiger charges were assigned to protein, and structural constraints were removed by 1,500 rounds of minimization runs (750 steepest descent followed by 750 conjugate gradients) with a step size of 0.02 Å, under ff03. rl force field (Pettersen et al., 2004).

### 2.7 Druggable and catalytic pocket detection

The filtered targets were subsequently examined for potential binding pockets by calculating the druggable score using the DoGSiteScorer programme (https://proteins.plus). Protein + contains several automated tools, including a pocket detection technique used to assess the druggability of protein cavities. To utilise Protein+, the target of interest must be in 3D format (.pdb). Consequently, structures obtained from SWISS-MODEL alongside MHOLline were employed in this phase. DoGSiteScorer was then applied to evaluate the druggability of these 3D structures, which provides the pocket residues and druggability scores (ranging from 0 to 1). A protein cavity with a score closer to one is considered to be a highly druggable protein cavity.

### 2.8 Retrieval of ligand libraries, molecular docking, and ADMET profile

To get druggable compounds with a Tanimoto threshold level of 60%, the ZINC database was queried for (n = 11,993) druggable lead molecules (Sterling and Irwin, 2015; Sterling and Irwin, 2015), additionally, compounds retrieved from the TCM database (Traditional Chinese Medicine) (http://ZINC.docking.org/) (n = 36,043) were also screened for top lead compounds selection through the MOE (Molecular Operating Environment) (Vilar et al., 2008). In the next step, partial charges were added, and their energies were reduced using an energy minimization method (default parameters). Following their binding energies, chemicals that had been docked were sorted in ascending order of their binding energies. All best structure conformations with the least amount of energy were selected. A docking study was conducted by using the MOE to transform the 3D structure of the proposed drug targets into receptor molecules for virtual screening. The entire docking procedure concluded with the selection of the best-docked chemical compounds, which were analyzed using the ligand interaction mode of MOE (Molecular Operating Environment) (Chemical Computing Group, Inc., 2013) to understand the interaction that contributed to the binding of the ligands (Vilar et al., 2008).

To find appropriate inhibitors, they must pass Lipinski’s drug-like test while still requiring the least amount of energy (Lipinski, 2000; Barker and Khossravi, 2001; Lipinski et al., 2001). We conducted ADME/Tox analysis on the top-scoring compounds using an ADMET prediction server (http://lmmd.ecust.edu.cn/admetsar2) (Yang et al., 2019) alongside the SwissADME server (http://www.swissadme.ch/) to validate further physicochemical characteristics of selected hits for further skin permeation values (Daina et al., 2017).

### 2.9 Molecular dynamics (MD) simulation of the predicted targets

Molecular dynamics (MD) simulation was used to study the ligand-receptor interactions over time, providing insights into particle movement and complex stability within the system. MD simulations rely on classical and Newtonian mechanics, including Molecular Mechanics/Quantum Mechanics (MM/QM) methods, among others. Using GROMACS v4.1.5 and the GROMOS 54a7 force field, simulations captured the dynamic behavior of these complexes (Páll et al., 2020; Michaud-Agrawal et al., 2011). A triclinic periodic boundary box with 10 Å extensions surrounded the protein structures, allowing ample space for solvation and minimization.

Using the PyMol program, the ligands were first saved in. mol format before being converted to. pdb format. The MD simulation process involved four stages: minimization (1,500 steps, including steepest descent and conjugate gradient to remove steric clashes), heating to the target temperature, NVT and NPT equilibration for temperature and pressure stabilization, and a 100 ns production run. The RESPA integrator managed motion integration and covalent bonds with hydrogen atoms (Ma et al., 2003). TIP3P water molecules and 0.15 M Na^+^ and Cl^−^ ions ensured system stability and charge neutrality (Jorgensen et al., 1983).

After the simulation, the root mean square deviation (RMSD), root mean square fluctuation (RMSF), solvent-accessible surface area (SASA), hydrogen bonds (H-bonds), and the radius of gyration (Rg) were calculated to assess stability and conformational behavior. Built-in GROMACS modules and XMgrace generated these outputs, which provide insights into the structural dynamics of the complexes (Michaud-Agrawal et al., 2011).

## 3 Results and discussion

### 3.1 Genome retrieval and identification of core genomes

**FIGURE 1:**
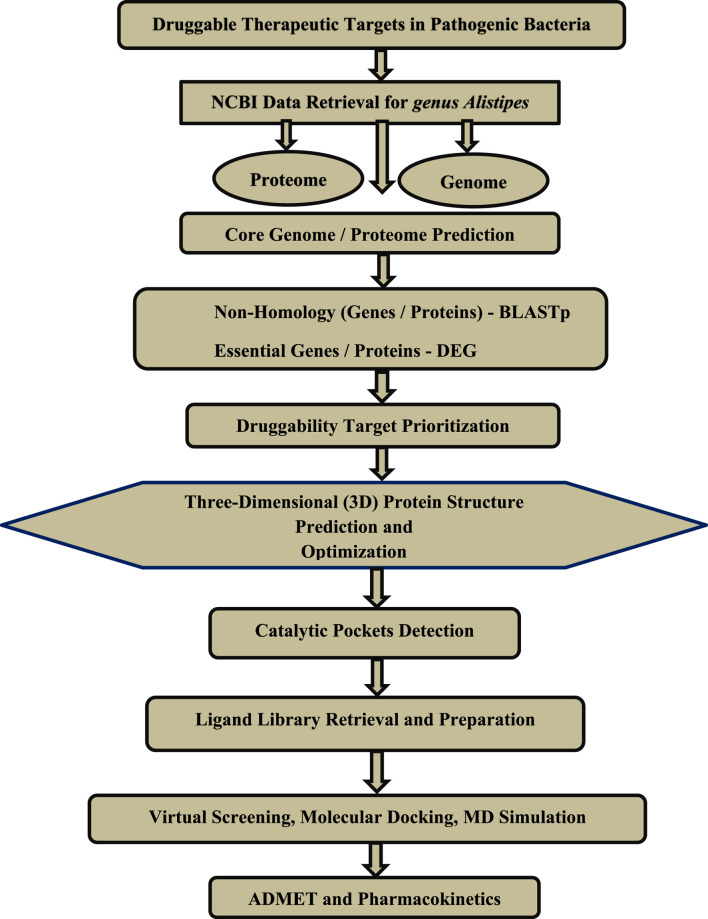
The general workflow of the step-by-step methodology followed in this study is based on subtractive genomics for the identification of druggable therapeutic targets.

**FIGURE 2:**
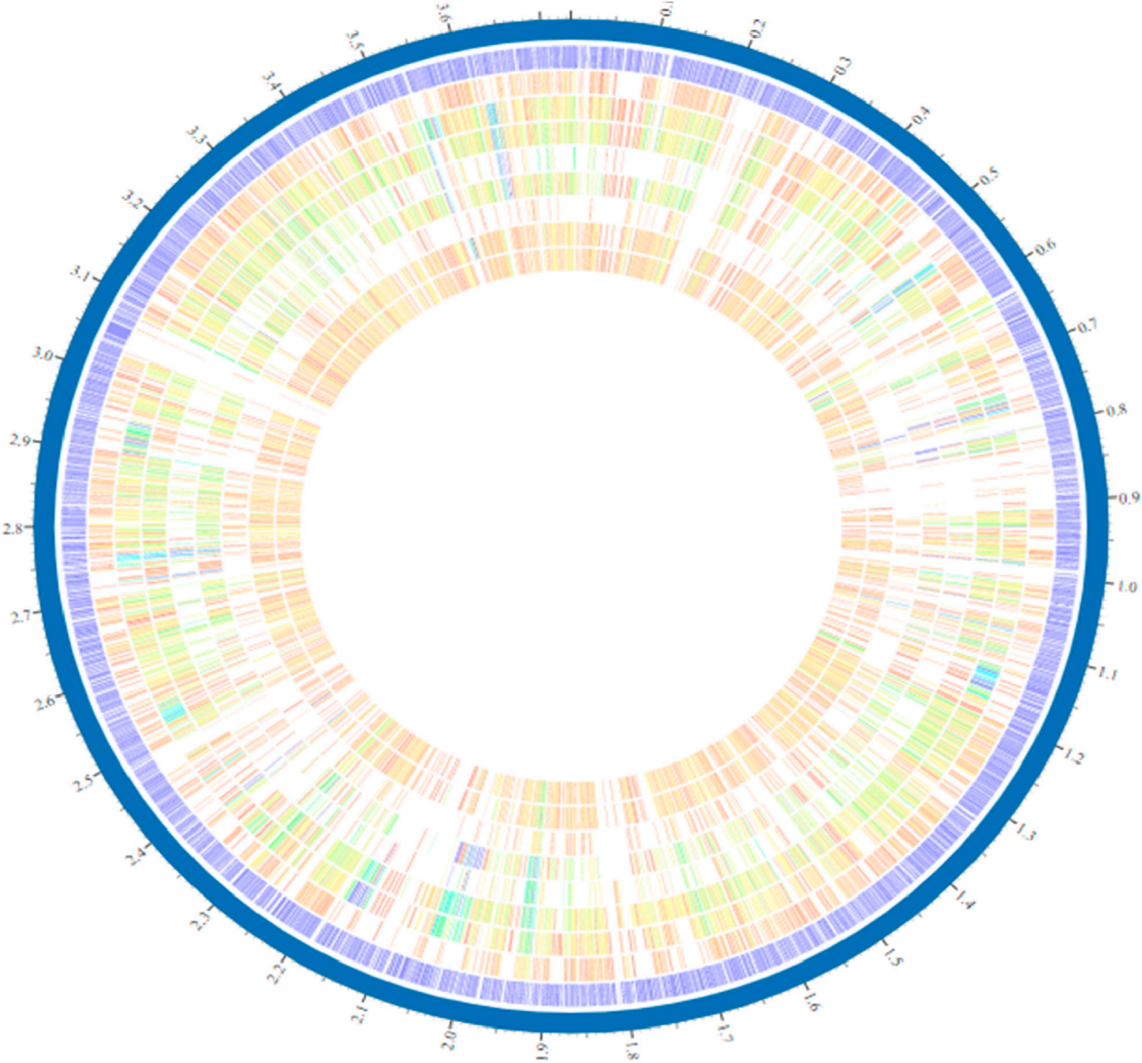
Circular genome representation of *Alistipes* generated through the PATRIC server. Among strains of the species Alistipes, varying hues and intensities indicate the existence or absence of distinct genes, genic islets, genomic islands, or other genetic elements.

The genome assembly and annotation report of the selected strains were checked using the NCBI (National Center for Biotechnology Information), a database resource that provides access to biomedical and genomic information. The complete genome sequence of *A. finegoldii* was obtained from the NCBI database and was randomly used as “the reference strain” to ensure the correctness of the results. To implement the subtractive genomics approach, only the complete genomes were selected for all *Alistipes* strains (Figure 1). The core genome was explored to discover pharmacological targets that were orthologs across all strains. For the most part, only the core genes of organisms, which are defined as the genes that are consistently present in all populations of an organism in all sorts of harsh conditions, were retracted. The core genome was discovered *via* the PATRIC program, and the overall number of genes discovered in the pangenome was 3,018, with 2,875 of them being non-redundant genes (Figure 2). After genome retrieval, the redundant gene removal was performed using a PATRIC application. This led to the identification of 143 redundant genes, and the remaining set was subjected to further genome subtraction analysis. Genome statistics like genome size, number of proteins, % GC content, bio-project information, and genome assembly data, among others, of all the selected strains are tabulated in Supplementary Table A.

### 3.2 Identification of non-host homologous and essential proteins

It is necessary to parse the file produced by the NCBI-BLASTp of the *A. finegoldii* core genome against the human genome. In total, 2,875 core genes were identified, and 2,571 proteins were found to be non-homologous with the human proteome. 344 genes were found to be host-homologous genes and were removed to avoid the resulting side effects. The BLASTp search was performed against prokaryotes, eukaryotes, and archaea for the host non-homologous proteins to determine the DEG essentiality (*e-value* = 10^-4^, *bit score* = 100 and sequence *identity* = ≥ 30%) (Luo et al., 2014). The results showed that out of the non-homologous set, 490 proteins are essential to *Alistipes finegoldi*. These steps are important for mining essential targets that are reportedly involved in performing vital cellular functions and avoiding cross-reactivity/binding of the drugs to undesired host protein sites.

### 3.3 Subcellular localisation and PPI for drug target prioritization

**FIGURE 3:**
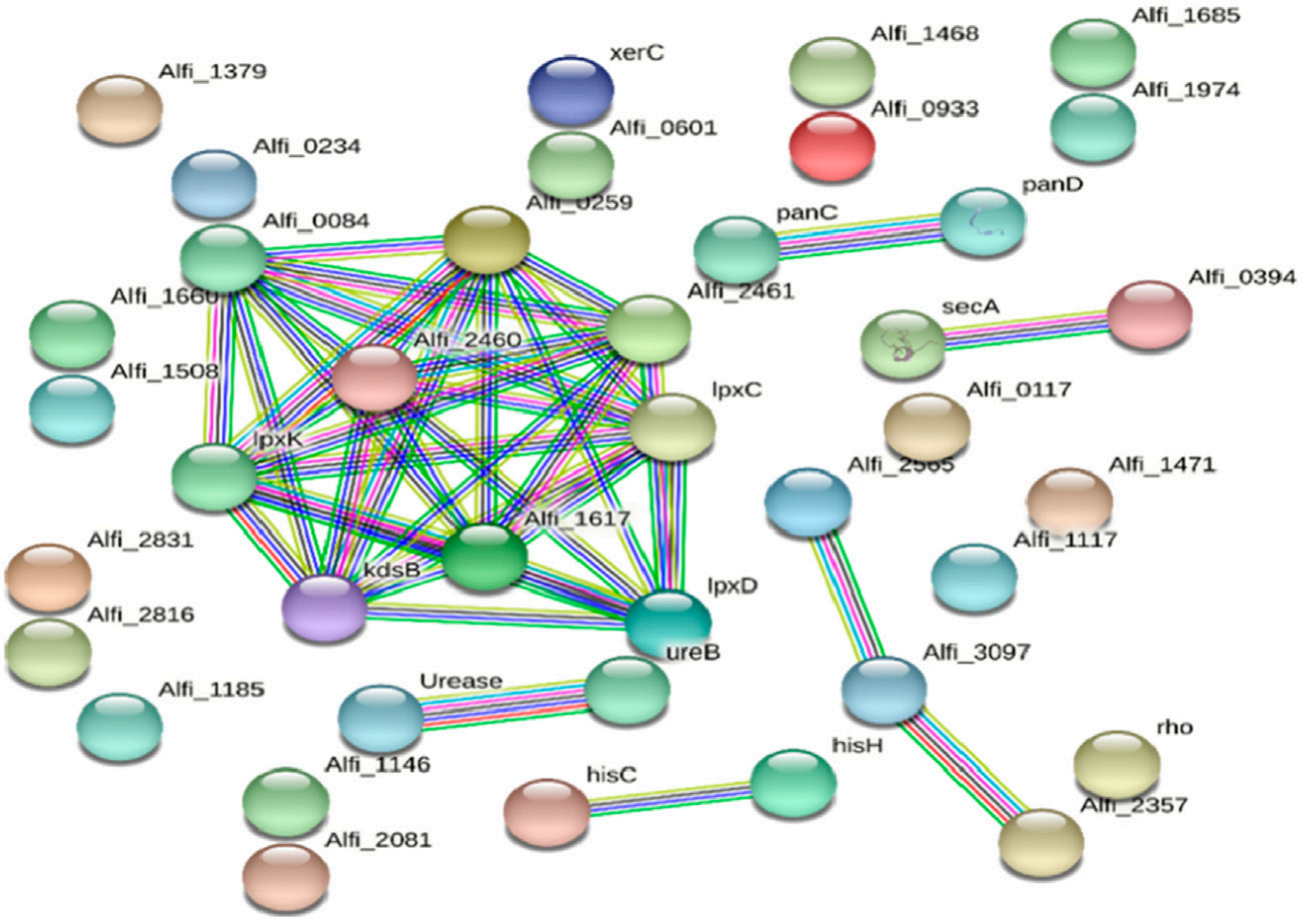
The STRING network of protein-protein interactions of the potential protein targets in *Alistipes*. The nodes and edges represent the proteins and their interactions, respectively, where the edge color represents the nature of the predicted interaction (experimentally curated or computationally predicted). The nodes are either shown as colored circles (query proteins and first shell of interactors) or white circles (second shell of interactors) and either empty circles (proteins of unknown 3D structure) or filled circles (3D structures are known or predicted). While line color reflects the type of interaction evidence and line width shows the strength of data support, the edges show both functional and physical protein interactions. Of the known interactions, those in purple have been determined experimentally, whereas those in cyan are from carefully selected databases. Gene neighborhood analyses are shown by green in expected interactions, gene fusion events by red, and gene co-occurrence by blue. The remaining relationships are navy blue = protein homology, black = co-expression, and olive = text-mining.

The essential targets were further processed for subcellular localisation prediction to refine the selection of potential drug targets, where 325 were cytoplasmic proteins, 150 were cytoplasmic-membrane proteins, 6 were inner-membrane proteins, and 1 was a periplasmic protein. Cytoplasmic proteins were selected as they are crucial for bacterial growth and metabolism and play essential roles in regulation, making them viable candidates for small-molecule inhibitors. Deciphering the protein-protein interactions network is very important in understanding the role of individual proteins and, thereby, in their classification and prioritisation as targets. After drug target prioritisation, a set of 39 proteins was submitted to the STRING database to identify hub proteins showing multiple interactions. The STRING database determines the interrelation between proteins, which is essential for proper functioning and gives detailed knowledge about proteins involved in single or multiple pathways. In total, nine proteins were shown to have numerous interactions and were thus classified as hub proteins and were considered for KEGG analyses (Figure 3).

**FIGURE 4:**
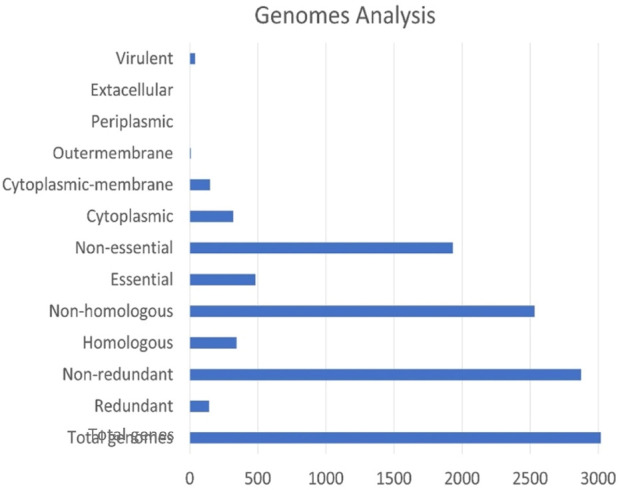
An overview of the screened proteins at the end of each subtractive genomics approach. The X-axis represents the total number of genes in all selected genomes, whereas the Y-axis represents the different steps of the subtractive genomics workflow towards compartmentalisation of genome portions based on their cellular function and localization.

The cytoplasmic proteins were prioritised based on properties like molecular weight, virulence factor, druggability score, and pathway analysis. All the predicted potential proteins were also screened against the virulence factor database (VFDB) (Chen et al., 2005b), which predicted them to be involved in pathogen virulence. The molecular weight of all proteins was less than 100 kDa, according to ProtParam; thereby, these molecules fulfill the Lipinski threshold. Figure 4 displays how the total number of core genes was reduced and allocated during the subtractive genomic approach. Theoretically, the Lipinski’s rule of five applies to oral active drugs, which make up the biggest class of medicinal compounds. A maximum of one violation is permitted. This rule was intended to be a general guideline for the chemist to take care of these factors to avoid any issues rather than to specifically exclude molecules or compounds that violated these principles. Nevertheless, it was still possible to biologically analyse the tested compounds. Lenacapavir, an HIV medication, and hepatitis C virus medications like ledipasvir, velpatasvir, and voxilaprevir are excellent examples. Nonetheless, the rule of five continues to offer medication designers helpful limits. For optimal oral bioavailability, the Veber’s rule restricts the surface area to 140Å and the number of rotatable bonds to 10. The GI absorption and impaired permeability of the chosen compounds of interest across the membrane’s bilayer would generally be affected by any medicine that deviates from both of these guidelines. While more rotatable bonds can improve solubility and drug absorption, they can also decrease permeability (Lipinski, 2000; Lipinski et al., 2001; Caminero Gomes Soares et al., 2023; Hartung et al., 2023).

### 3.4 Metabolic pathway analyses via KEGG

**FIGURE 5:**
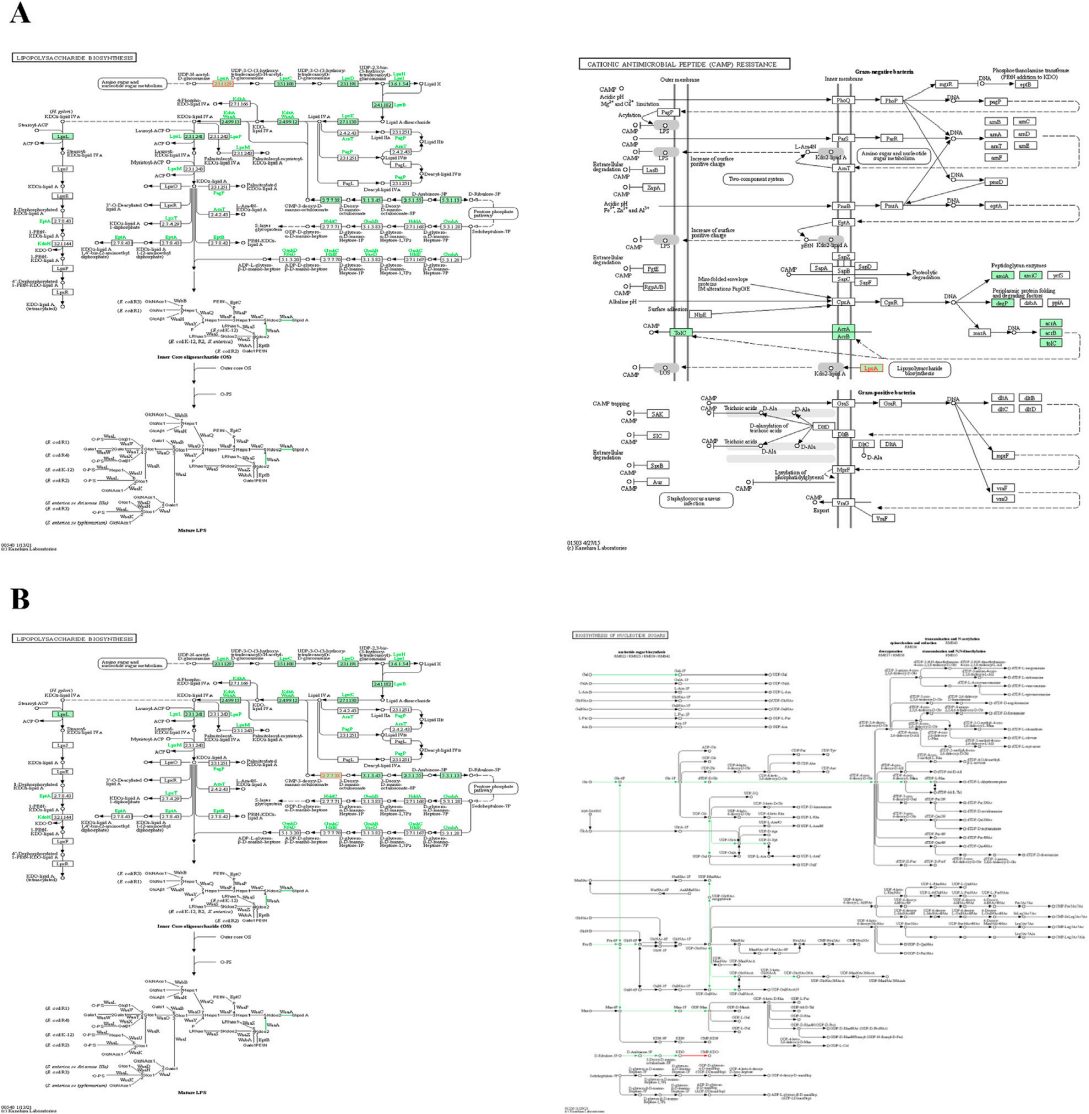
Important pathways of final targets **(A)** Alfi_0084 and **(B)** Alfi_2459. For a detailed description, the readers are referred to the online KEGG database for the genus *Alistipes*. Metabolites are shown by nodes, metabolite class is indicated by shape, and reactions are represented by lines.

The query nine proteins were submitted to a pathway analysis using the KEGG database (Kanehisa et al., 2017). It is quite evident from the obtained results that most of the targets were engaged in multiple metabolic pathways, such as lipopolysaccharides biosynthesis and fatty acid biosynthesis, among others. In contrast, only one protein was involved in resistance pathways, such as the cationic antimicrobial peptide resistance pathway (CAMP) (Table 1; Figure 5).

**TABLE 1:** Specific drug target prioritization criteria induced and functionally annotated for 9 non-host homologous proteins. Each identifier corresponds to a protein locus tag assigned by NCBI rather than specific protein names, which are given in a separate column.

Protein locus tags	Protein name (GenBank)	Protein function	KEGG pathways
Alfi_0084	1. acyl-acyl-(acyl-carrier-protein) --UDP-N-acetylglucosamine O-acyltransferase 2. UDP-N-acetylglucosamine acyltransferase	involved in the biosynthesis of lipid A, a phosphorylated glycolipid that anchors the lipopolysaccharide to the outer membrane of the cell	1. Afd00540 Lipopolysaccharide (LPS) biosynthesis 2. Afd01100Metabolic pathways 3. Afd01503 Cationic antimicrobial peptide (CAMP) resistance
Alfi_0259	3-deoxy-D-manno-octulosonic-acid transferase	Lipopolysaccharide (LPS) biosynthesis. Catalyzes the transfers of 3-deoxy-D-mannoz-octulosonatez (Kdo) residues(s) from CMP- Kdo to lipid IV(A), the tetraacyldisaccharide-1,4′-bisphosphate precursors of lipid A	1. Afd00540 LPS biosynthesis 2. Afd01100 Metabolic pathways
Alfi_1617	lipid-A-disaccharide synthase	Condensation of UDP-2,3-diacylglucosamine and 2,3- diacylglucosamine-1-phosphate to form lipid A disaccharide, a precursor of lipid A, a phosphorylated glycolipid that anchors the lipopolysaccharide to the outer membrane of the cell	1. afd00540 LPS biosynthesis 2. afd01100Metabolic pathways
Alfi_2460	1.1. 3-Deoxy-8-phosphooctulonate synthase 2-dehydro-3-deoxyphosphooctonate aldolase (KDO 8-P synthase)	Belongs to the KdsA family	1. Afd00540 LPS biosynthesis 2. Afd01100 Metabolic pathways
Alfi_2461	1. KpsF/GutQ family protein 2. Arabinose-5-phosphate isomerase	Belongs to the SIS family. GutQ/KpsF subfamily	1. afd00540 LPS biosynthesis 2. afd01100Metabolic pathways
Alfi_2459 (*KdsB*)	3-deoxy-D-manno-octulosonate cytidylyltransferase (CMP-KDO synthetase)	Activates KDO (a required 8-carbon sugar) for incorporation into bacterial lipopolysaccharide in Gram-negative bacteria	1. afd00540 LPS biosynthesis 2. afd01100Metabolic pathways
Alfi_0083 (lpxC)	1. Beta-hydroxyacyl- (acyl carrier protein) dehydratase FabZ 2. UDP-3-O-[3-hydroxymyristoyl] N-acetylglucosamine deacetylase/3-hydroxyacyl-[acyl-carrier-protein] dehydratase	Catalyzes the hydrolysis of UDP-3-O-myristoyl-N- acetylglucosamine to form UDP-3-O-myristoylglucosamine and acetate, the committed step in lipid A biosynthesis	1. Afd00061 Fatty acid biosynthesis 2. Afd00540 LPS biosynthesis 3. Afd01100 Metabolic pathways 4. Afd01212 Fatty acid metabolism
Alfi_0445 (lpxD)	UDP-3-O-(3-hydroxymyristoyl) glucosamine N-acyltransferase	Catalyzes the N-acylation of UDP-3-O-acylglucosamine using 3- hydroxyacyl-ACP as the acyl donor. Is involved in the biosynthesis of lipid A, a phosphorylated glycolipid that anchors the lipopolysaccharide to the outer membrane of the cell	1. Afd00540 LPS biosynthesis 2. Afd01100 Metabolic pathways
Alfi_2490 (lpxK)	1. Lipid-A-disaccharide 1. Lipid-A-disaccharide kinase 2. Tetraacyldisaccharide 4′-kinase	Transfers the gamma-phosphate of ATP to the 4′-position of a tetraacyldisaccharide 1-phosphate intermediate (termed DS-1-P) to form tetraacyldisaccharide 1,4′-bis-phosphate (lipid IVA)	1.afd00540 Lipopolysaccharide biosynthesis 2.afd01100 Metabolic pathways

### 3.5 Proteins 3d structure modeling and druggability assessment

**FIGURE 6:**
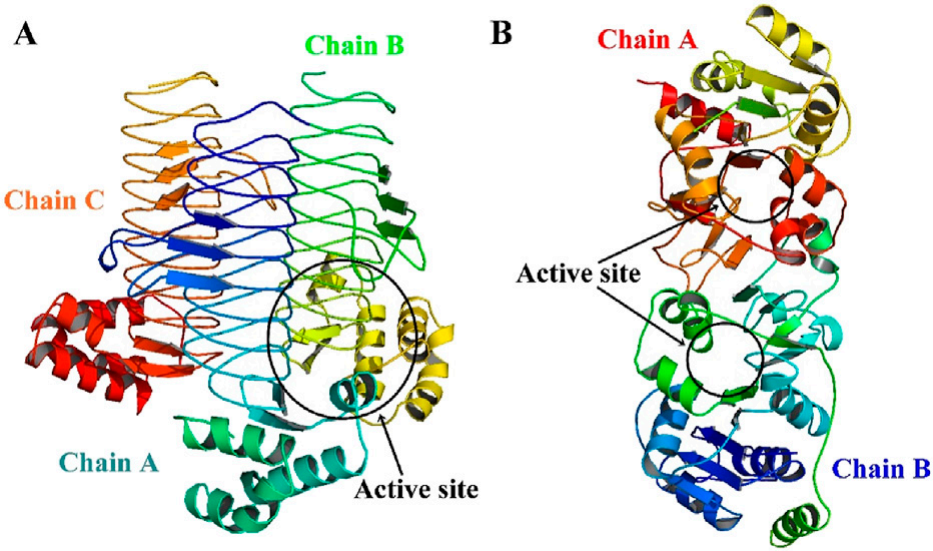
3D structures of target proteins generated using SWISS-MODEL **(A)** *LpxA* (Alfi_0084) and **(B)** *KdsB* (Alfi_2459).

From the set of nine proteins, only two cytoplasmic proteins were chosen as prospective therapeutic targets based on the metabolic pathways they are involved in and the % identity with their respective templates (RCSB-PDB, ≥25%). The 3D structure availability of a protein is the starting point for CADD analysis. The structure of identified druggable receptor proteins, i.e., the *LpxA* and *KdsB*, were generated through the SWISS-MODEL (Figure 6). The information on the active site residues was retrieved from the respective template structures for each putative target and was useful during the docking step for best inhibitor selection (Table 2). After cross-checking the stereochemical properties, the best 3D model structure was selected. Model 1 from the SWISS-MODEL was subjected to further analysis based on good physicochemical and quality measures. Besides significant coverage, it showed strong stereochemistry with no residue in the disallowed region and the lowest Z score. Energy minimisation was carried out to relax the structure and remove the steric clashes of the side chain. The physicochemical and Ramachandran properties of both models, where maximum residues are present in the most favored regions, are given in Table 3.

**TABLE 2:** Detailed description of 3D structures and active site residues of final 2 target proteins.

S. No.	NCBI Accession Number/s	Protein Name/s	Template	Identity	Active site residues
*LpxA* (Alfi_0084)					
1	WP_014774337.1	acyl-acyl-(acyl-carrier-protein) --UDP-N-acetylglucosamine O-acyltransferase	5DEP	35.69%	LEU65, GLN64, ILE130, ILE148, PHE166, ASN194, ARG201
*KdsB* (Alfi_2459)					
2	WP_014776009.1	3-deoxy-D-manno-octulosonate cytidylyltransferase (CMP-KDO synthetase)	3DUV	39.59%	LYS74, GLN98, ARG157, HIS185, GLU 214, GLN 215

**TABLE 3:** Stereochemical and Ramachandran properties of comparative homology structures.

Targets	Most favored	Allowed	Disallowed	ERRAT	VERIFY3D	Bad contacts	G factors
*LpxA* (Alfi_0084)	92.75%	5.93%	1.32%	92.25	92.16 (pass)	0	−0.03
*LpxA* (Alfi_2459)	95.14%	8%	1.62%	92.79	82.73 (pass)	0	0.09

**FIGURE 7:**
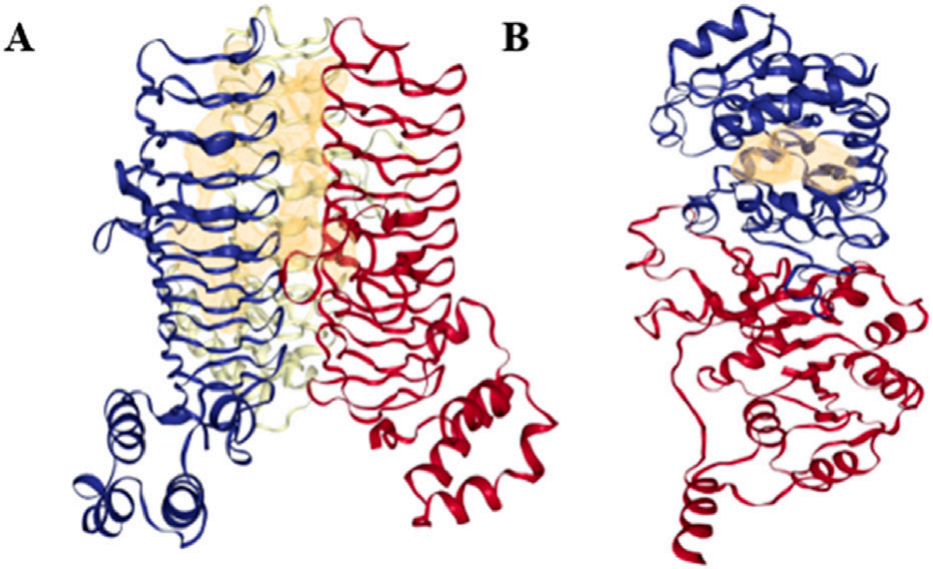
Highly druggable pockets of the final targets **(A)** *LpxA* (Alfi_0084), **(B)** *KdsB* (Alfi_2459). Only one highly druggable pocket is shown for each target protein in yellow mesh form as predicted by the DoGSiteScorer (druggable pocket ≥0.6, highly druggable pocket ≥0.8).

The DoGSiteScorer was used to establish a link between the drug-binding ability of the predicted targets based on their binding pocket structure and activity. The DoGSiteScorer does functional characterisation, druggability estimate, and automatically predicts pockets and sub-pockets in a target protein’s 3D structure. A protein that exhibits the highest contact affinity with a drug molecule is said to be highly druggable. On a scale of 0–1, the druggability measurement is scored as follows: ≥0.6 for medium-to-high druggable proteins and ≥0.8 for highly druggable proteins. Even though a protein of interest may have multiple anticipated druggable pockets, docking analyses typically only take into account the highly druggable pockets. The druggable pockets with their corresponding druggability statistics were identified for both *LpxA* and *KdsB* (Alfi_0084 and Alfi_2459), and are given in Table 4, only the highly druggable pockets are shown in Figure 7.

**TABLE 4:** DoGSiteScorer pockets and druggability scores for the Alfi_0084 (*LpxA*) and the Alfi_2459 (*KdsB*). Only the highly druggable pockets (bold) were considered further as the best binding sites.

*LpxA(*Alfi_0084) pockets	Volume Å^3^	Surface Å^2^	Druggability score	Simple score
P_0084_2	**802.99**	**746.69**	**0.86**	**0.5**
P_0084_1	2006.14	2,379.46	0.81	0.63
P_0084_3	148.14	334.27	0.42	0.0
P_0084_4	147.92	290.53	0.33	0.0
*KdsB (*Alfi_2459) Pockets				
P_24559_2	**475.56**	**844.92**	**0.77**	**0.31**
P_24559_1	628.35	745.17	0.63	0.37
P_24559_4	226.71	410.35	0.52	0.03
P_24559_3	235.12	252.23	0.51	0.1
P_24559_5	206.15	502.74	0.35	0.12

The best binding sites are the highly druggable pockets with different druggability parameters (in bold) as predicted by the online DoGSiteScorer platform.

### 3.6 Molecular docking and ADMET profiling of top compounds

A total of 11,993 ZINC compounds were screened against two targets using the MOE docking pipeline. Based on known ligands of the template structures and their active site residues reported in the literature, lead compounds were identified for *LpxA* (Alfi_0084) and *KdsB* (Alfi_2459). The active site residues from the template structures were cross-validated with the druggable cavities identified using DoGSiteScorer and further analyzed using MOE and PyMOL tools to ensure precise ligand placement.

After uploading and preparing the receptor/protein models, MOE provided multiple cavity selection options, allowing us to confirm that docking was performed in the most biologically relevant and druggable sites. This step ensured that docking occurred within functionally significant binding pockets while also aligning with the druggability predictions. To maximize inhibitor screening, a second compound library from Traditional Chinese Medicine (TCM, n = 36,043) was also evaluated.

The top hits for each receptor were docked, and the five best compounds from each library were further analyzed based on binding affinity, hydrogen bonding interactions, and structural orientation within the receptor’s active sites. The docking poses were carefully examined in MOE to ensure that the best-ranked molecules occupied functionally critical residues, reinforcing the accuracy of the docking process (Table 5).

**TABLE 5:** Docking results of inhibitors arranged in descending order with corresponding binding affinities within the *LpxA* (Alfi_0084) and the *KdsB* (Alfi_2459) binding sites.

*LpxA* (Alfi_0084)					
TCM compounds	Binding affinity	Hydrogen bond interactions	ZINC 12k Compounds	Binding affinity	Hydrogen bond interactions
ZINC85530940	−10.2535	His153, Val152, Ser133, Leu69, Gln67	ZINC05161112	−6.9723	Gly166, Gly148, Ala135
ZINC95919154	−10.1223	Gly148, Gln154, Val152	ZINC06507895	−6.89379	Asn130, Gln67, Ser133
ZINC85505096	−10.1162	Gly166, Gln67, Asp68, Ala135	ZINC58356220	−6.86195	Val152
ZINC95913537	−10.0993	Asn130, Arg198, Arg181, Asp68, Ser137, Gly148, Gln154	ZINC79002834	−6.85966	Gln67
ZINC85645304	−10.0465	Asn130, Arg197, Arg198, Ala135, Gln165, His153, Gln154	ZINC77524163	−6.82012	Ser133, Val152
Colistin	−8.0146	Asp68, Lys70, Met111, Gly147			
CHIR-090	−8.3480	Gly148, His153, Gln154, Gln172, Thr184			
*KdsB* (Alfi_2459)					
ZINC95911713	−10.0063	Lys72, Arg77, Gln96, Glu99, Glu210	ZINC05566415	−37.1697	Lys72, Asp76, Glu204
ZINC95918704	−9.84735	Pro8, Arg10	ZINC05557850	−36.861	Ser208, Glu210
ZINC95912877	−9.83038	Pro8, Gln96, Ser208, Glu210	ZINC05517668	−30.278	Lys72, Ser208, Gln211
ZINC85648570	−9.59031	Arg10, His71, Arg77	ZINC05669511	−29.8498	Arg158, Glu210
ZINC95913839	−9.54473	Pro8, His71, Gly74, Arg77, Gln96, GluA210	ZINC05731403	−27.9232	Lys72, Ser208
Colistin	−8.0343	Arg10, Lys72, Arg158, Ser208, Glu210			
CMP-KDO	−5.2954	Glu99, Pro143, Asp234			

**FIGURE 8:**
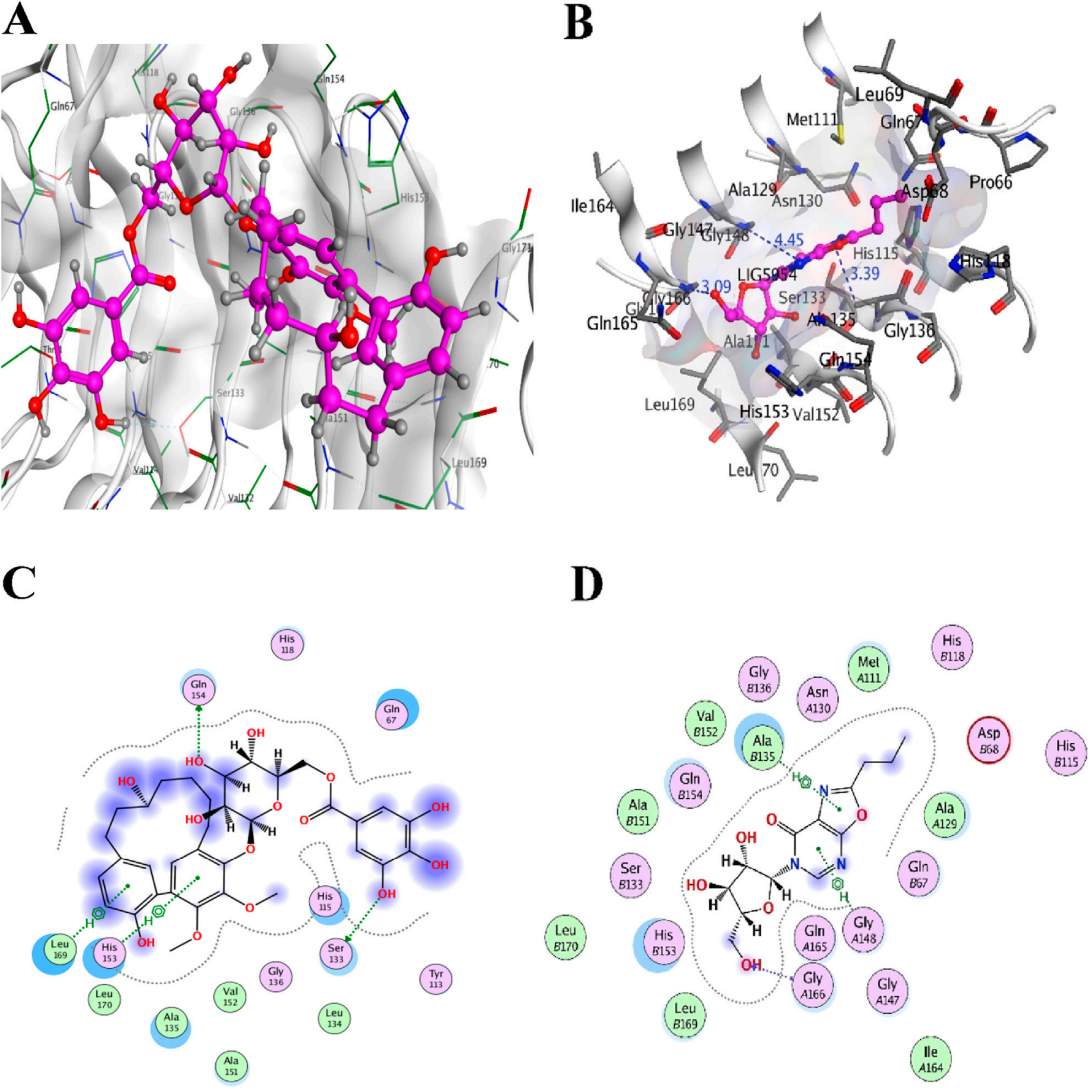
Top two inhibitors, each from TCM and ZINC libraries, respectively, are docked in the druggable cavity of the *LpxA*, representing hydrogen bond interactions **(A, C)** as 3D and 2D representation for ZINC85530940 and **(B, D)** as 3D and 2D representation for ZINC05161112).

For convenience and simplicity, only the *LpxA* and *KdsB* terms will be used hereafter for the two identified targets. The ligand confirmation of the five top compounds from each library was performed according to the binding affinities with the receptor targets, the *LpxA* and the *KdsB*. The modeled structures of the *LpxA* and the *KdsB* which are active in lipopolysaccharide’s metabolism and other critical pathways were docked against five TCM hits with scores −10.2535 kcal/mol, −10.1223 kcal/mol, −10.1162 kcal/mol, −10.0993 kcal/mol and −10.0465 kcal/mol and −10.0063 kcal/mol, −9.84735 kcal/mol, −9.83038 kcal/mol, −9.59031 kcal/mol, −9.54473 kcal/mol, respectively. Amongst the ZINC12k library, the top hits exhibited scores of −6.9723 kcal/mol, −6.89379 kcal/mol, −6.86195 kcal/mol, −6.85966 kcal/mol, −6.82012 kcal/mol, and −37.1697 kcal/mol, −36.861 kcal/mol, −30.278 kcal/mol, −29.8498 kcal/mol, −27.9232 kcal/mol for the LpxA and the KdsB receptors. Notably, the stability of the complexes in terms of the binding energy scores for the ZINC12k componds was observed as the lowest one, ranging between −27 kcal/mol and 37 kcal/mol. (Table 5; Figures 8; Figure 11).

**FIGURE 9:**
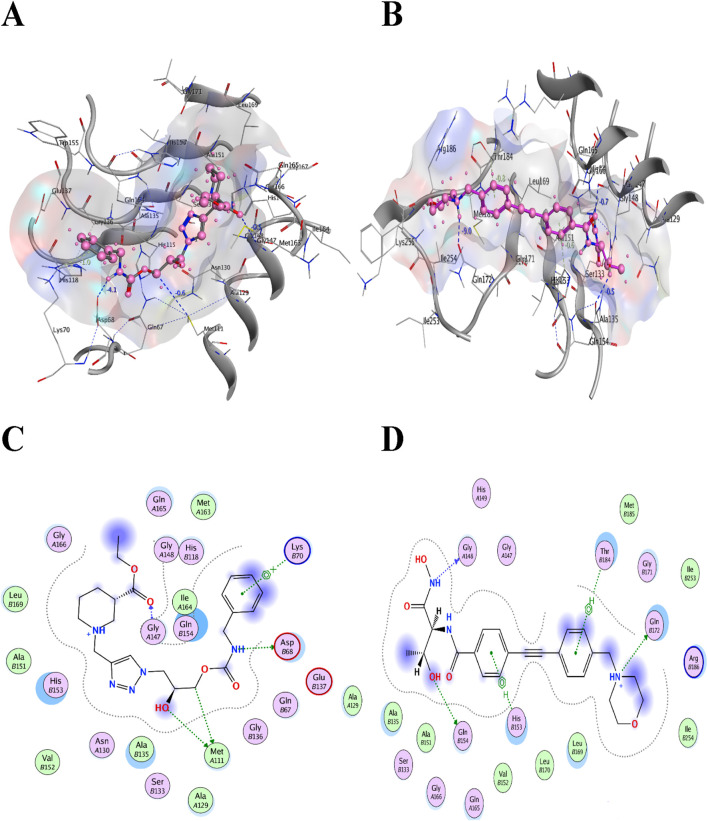
The two commonly used potent antibiotics are docked in the druggable cavity of the *LpxA*, representing hydrogen bond interactions **(A, C)** as 3D and 2D representation for Colistin and **(B, D)** as 3D and 2D representation for CHIR-090) and binding affinities of −8.0146 for Colistin and −8.3480 for CHIR-090, respectively.

**FIGURE 10:**
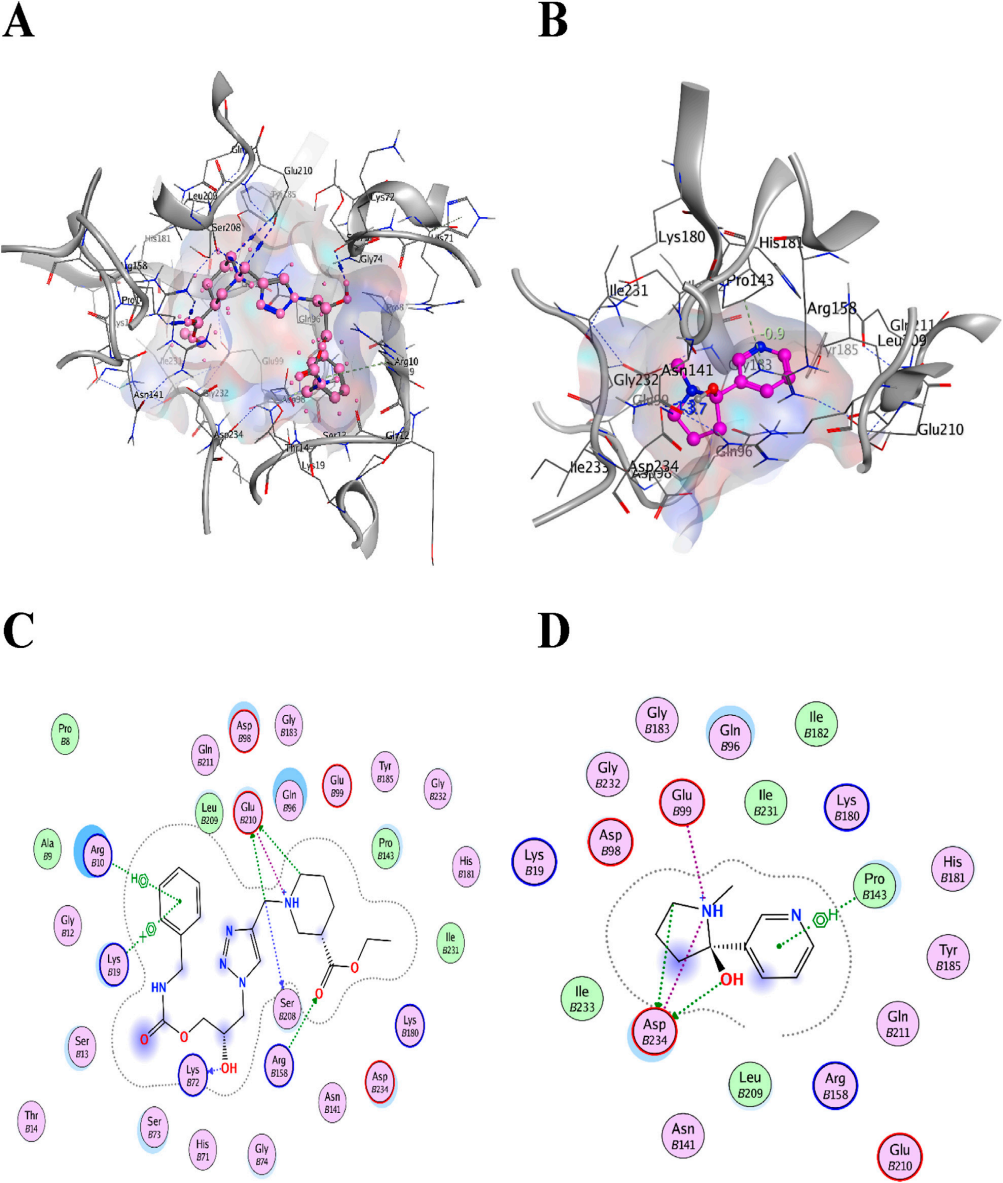
The two commonly used potent antibiotics are docked in the druggable cavity of the *KdsB*, representing hydrogen bond interactions **(A, C)** as 3D and 2D representation for Colistin and **(B, D)** as 3D and 2D representation for CMP-KDO) and binding affinities of −8.0343 for Colistin and −5.2954 for CMP-KDO, respectively.

Colistin and CHIR-090 are the reported potent inhibitors of the newly identified target *LpxA* of *Alisipes*, whereas Colistin again and CMP-KDO are the reported potent inhibitors of *KdsB.* The 2D/3D structures of these compounds were retrieved from the public databases and were docked as the reference compounds in the druggable cavities of the two target proteins. The number of H-bonds and the interacting residues, the S energy scores of the inhibitor-bound complexes and their 2D and 3D structural information are presented (Table 5; Figures 9; Figure 10). The binding affinities of the top TCM compounds are higher than the reference antibiotics with varying numbers and types of amino acid residues in the druggable cavities of both targets. However, the reference compounds exhibited higher affinities towards the druggable targets than the top ZINC compounds. On the other hand, the docked reference compounds for the *KdsB* have the lowest binding energy scores than the top compounds of both the TCM and ZINC databases. Again, the nature and number of the residues of the druggable cavities that made Hydrogen bond interactions remained different; in some cases, even higher numbers of interactions were observed for the reference antibiotic inhibitors (Table 5).

Further, the physicochemical properties of the top hits were checked, including the Lipinski rule of 5 (LR-5), a prime condition to fulfill the rational drug design, and the bioactivity scores for putative drugs of oral use. The ZINC compounds revealed no violations for the LR-5, i.e., no more than five hydrogen bond donors, no more than ten hydrogen bond acceptors, logP (partition coefficient) not more than five, rotatable bonds less than 10, polar floor location not extra than 140 and a molecular weight less than 500 g/mol. The Log Po/w information are also included in Table 6 for all selected inhibitors, where the consensus Log Po/w value of compound ZINC95911713 is 6.53, which violates the LR-5 somehow, yet it can be observed that the consensus Log Po/w values of other three best hits from ZINC and TCM libraries are much lower than 3.5. However, the TCM compound ZINC85530940 exhibited three violations of the Lipinski rule and one of the Veber rule, even as ZINC95911713 confirmed violations for each rule. Different analyses carried out for the top hit compounds are shown below in Table 6.

**TABLE 6:** Physicochemical characteristics (ADMET profile) of the final selected compounds.

Molecule	Formula	MW^$^ (g/mol)	RB^$$^	HBA^$$$^	HBD^%^	TPSA^%%^ (Å^2^)	BS^b^	PA^**^	LogP_o/w_^**^	SA^***^	SIC^c^	Log kp	LV^cc^	VV^ccc^
ZINC85530940	C34H40O14	672.67	8	14	8	225.06	0.17	1	1.85	7.76	Moderately soluble	−8.36 **(cm/s)**	3	1
ZINC95911713	C40H75NO9	714.02	33	9	7	168.94	0.17	0	6.53	8.24	Poorly soluble	−3.84 **(cm/s)**	2	2
ZINC05161112	C13H17N3O6	311.29	4	8	3	130.84	0.55	0	−0.40	4.47	Soluble	−8.45 **(cm/s)**	0	0
ZINC05566415	C7H15N5S	201.29	3	1	4	92.57	0.55	0	0.31	3.04	Soluble	−7.48 **(cm/s)**	0	0

^a^ **MW:** Molecular Weight ^**$$**^ **RB:** Rotatable Bonds ^**$$$**^ **HBA:** H-bond Acceptors.

^%^HBD: H-bond Donor ^%%^TPSA: topological polar surface area.

^b^ BS: Bioavailability Score **PA: PAINS, Alerts ***SA: synthetic accessibility.

^c^ SIC: Silicos-IT, Class ^##^LV: Lipinski Violations ^###^VV: veber violations.

Among the top five hits from TCM and ZINC12K libraries, ZINC85530940 and ZINC05161112 for *LpxA* and ZINC95911713 and ZINC05566415 for *KdsB* were selected, respectively, based on their least binding energies, the number of hydrogen bonds and an adequate orientation within the active site cavity. The goal of the study was to screen two different compound databases using a similar approach to identify potential inhibitors. The large differences in binding affinity predictions were the top compounds from TCM were the best candidates for *LpxA*, and the best compounds from ZINC 12 were the best for *KdsB*. In addition, since there is no reference for a binding affinity threshold, remarkable differences in the binding affinity of these inhibitors were observed. The interaction of inhibitors with their target binding sites could be attributed in terms of binding affinities, i.e., high affinities in inhibitor-receptor complexes result from greater attractive forces between the ligand and its receptor and *vice versa*.

**FIGURE 11:**
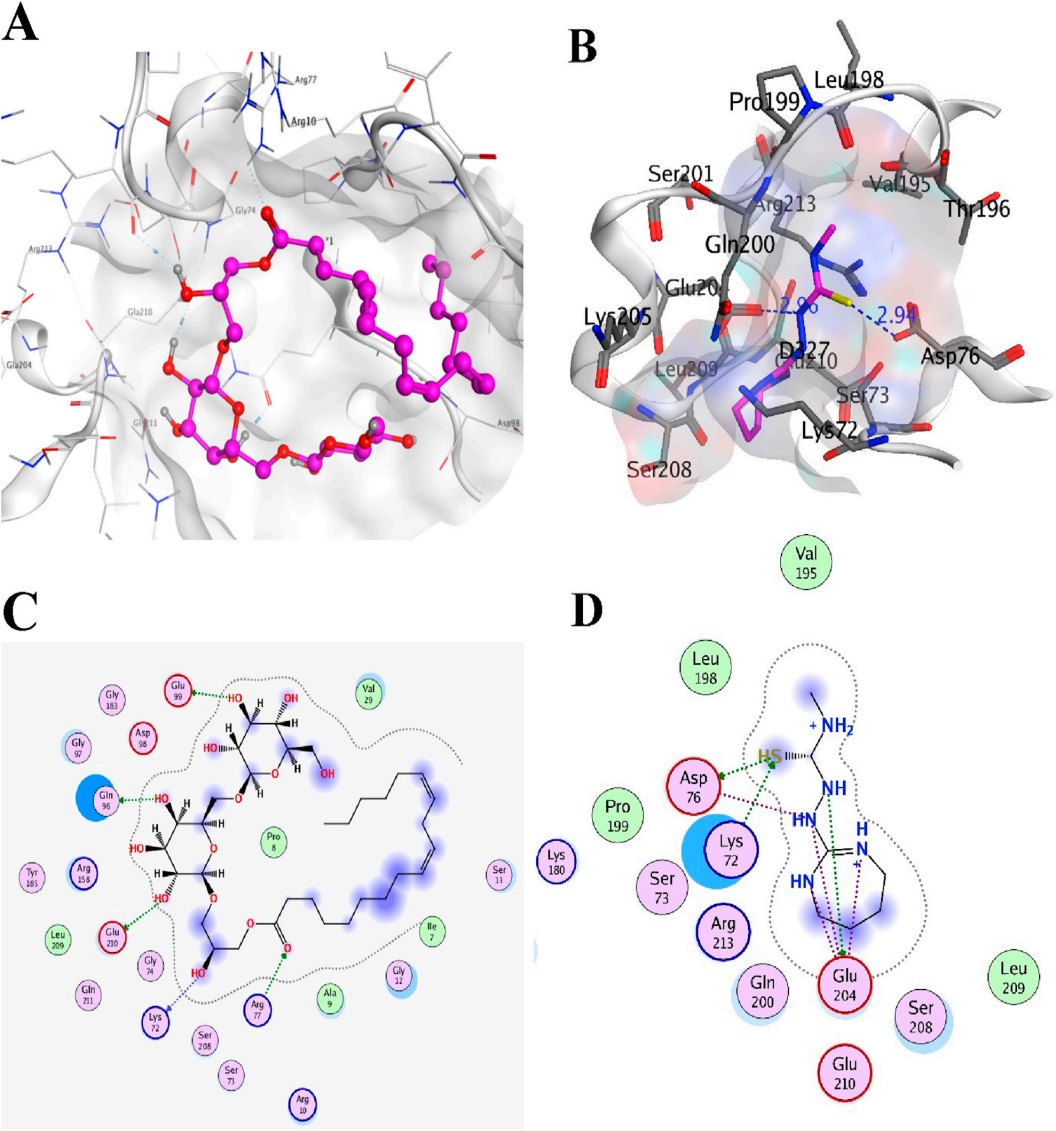
Top two inhibitors, each from TCM and ZINC libraries, respectively, are docked in the druggable cavity of the *KdsB*, representing hydrogen bond interactions **(A, C)** as 3D and 2D representation for ZINC95911713 and **(B, D)** as 3D and 2D representation for ZINC05566415).

According to the results, the top hits possess good bioactivity and drug-like properties, as shown in Table 6. The identified best docking pose revealed the most interacting residues in the active sites of the *LpxA* and the *KdsB*. The pose view within the druggable pockets showing interactions between the selected compounds and their respective individual targets are shown in Figures 8; Figure 11.

### 3.7 Molecular dynamic simulation

The most fundamental element associated with the function of proteins is their conformational dynamics. Functional information of protein molecules is encrypted in its structure. To unravel its functional variability, a comprehensive understanding of the structure is needed. Here, the MD simulation was performed as per the available computational facilities at the time this study was conducted to explore the conformational aspect of protein-ligand interactions and to evaluate the stability of the homology model and enzyme inhibitor complex. Data analysis like root mean square deviation (RMSD), root mean square fluctuation (RMSF), radius of gyration (Rg) and β-factor values, solvent-accessible surface area (SASA), and Binding energies were used to determine the conformational changes and stability index of secondary structure elements of the simulated complexes.

#### 3.7.1 Root mean square deviation (RMSD)

**FIGURE 12:**
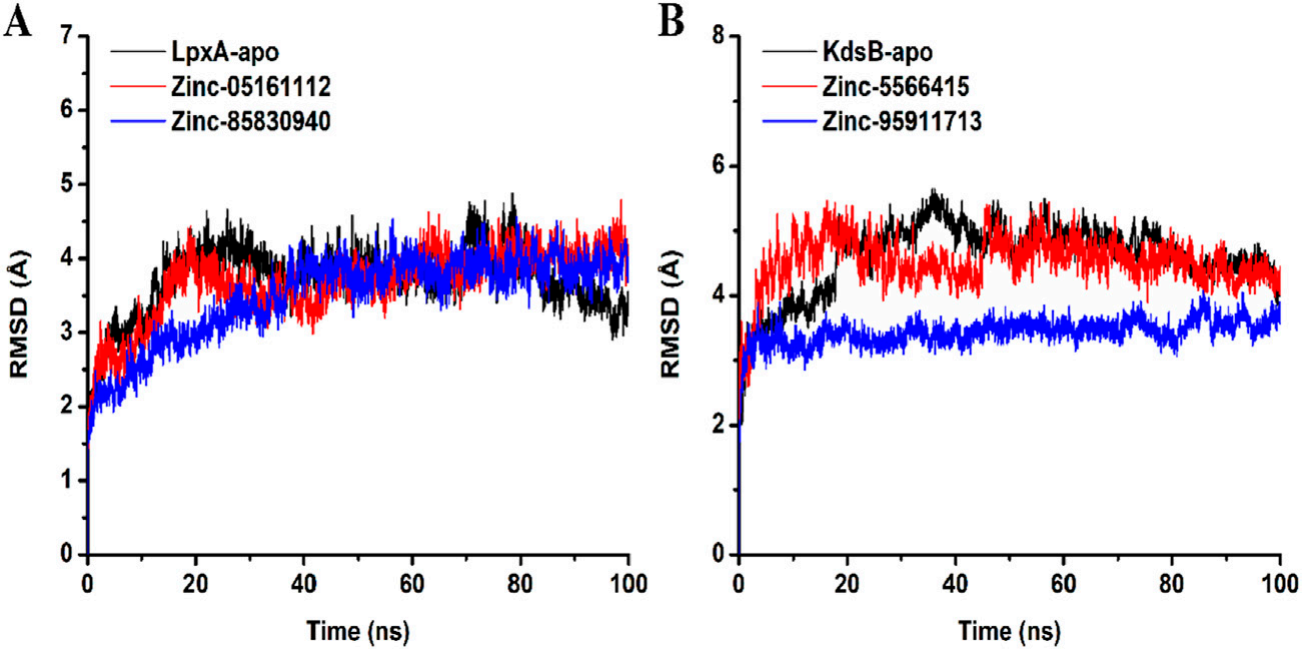
RMSD plot of the *LpxA* **(A)** and the *KdsB* **(B)** for apo and ligand-bound complex over 100ns simulation run.

RMSD explains the backbone analysis and Cα atoms dynamics over some time of docked protein (Figure 12). For both the *LpxA* and the *KdsB*, minor fluctuations were observed initially at the start of the simulation, but as the simulation proceeded, stability was observed for the apo and the docked complexes. The average RMSD value for the *LpxA*-apo was 3.7Å with an SD of 0.4; for ZINC05161112, the RMSD value was 3.67Å with an SD of 0.48, and for ZINC85830940, the value stabilized at 3.49 Å with an SD of 0.58. The average RMSD value for the *KdsB* was 4.59Å with an SD of 0.56; for ZINC5566415, the value was 4.51Å with an SD of 0.40, while ZINC95911713 exhibited an RMSD of 3.4 Å with an SD of 0.2, exhibiting more stability than any other docked or apoprotein structure. Overall, the pattern of the RMSD graph does not support any major domain shifts within the structural framework of the protein-ligand complex. The placement of ligands was well-complemented within the binding site during the simulation and stabilized the protein significantly Figure 12.

#### 3.7.2 Root mean square fluctuations (RMSF)

**FIGURE 13:**
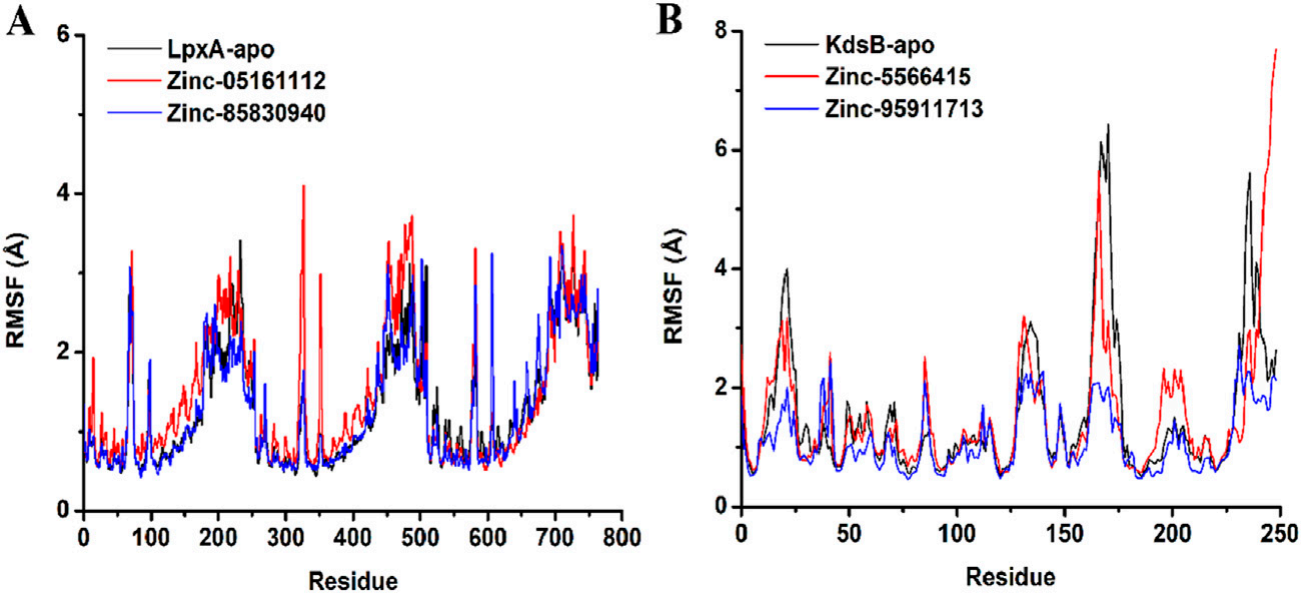
RMSF plot of the *LpxA* **(A)** and the *KdsB* **(B)** for apo and ligand-bound complex over 100ns simulation run.

Structure flexibility and fluctuation of Cα residues over time were observed by the RMSF graph analysis. The RMSF values of the apo *LpxA* protein calculated were highly in line with the RMSF values obtained for the docked complexes. Some major fluctuations were observed for ZINC05161112 at 327th residue having 4.1Å and 352nd residue exhibiting 2.9 Å that was unique to this docked complex. For ZINC85830940, residue 607th exhibited a 3.2Å RMSF value that was stable for the other docked and apoprotein. The RMSF of the remaining residues depicted comparably more compactness of the ligand-bound protein than the apo *LpxA* protein. For the *KdsB* protein, the RMSF values were much lower, depicting more compactness than the apoprotein. The loop region of the ZINC95911713-bound *KdsB* protein at the N terminal region exhibited more fluctuations. However, overall, the RMSF graph represented much more compactness in the ligand-bound *KdsB* protein compared to the apoprotein (Figure 13).

#### 3.7.3 β-Factor analysis

**FIGURE 14:**
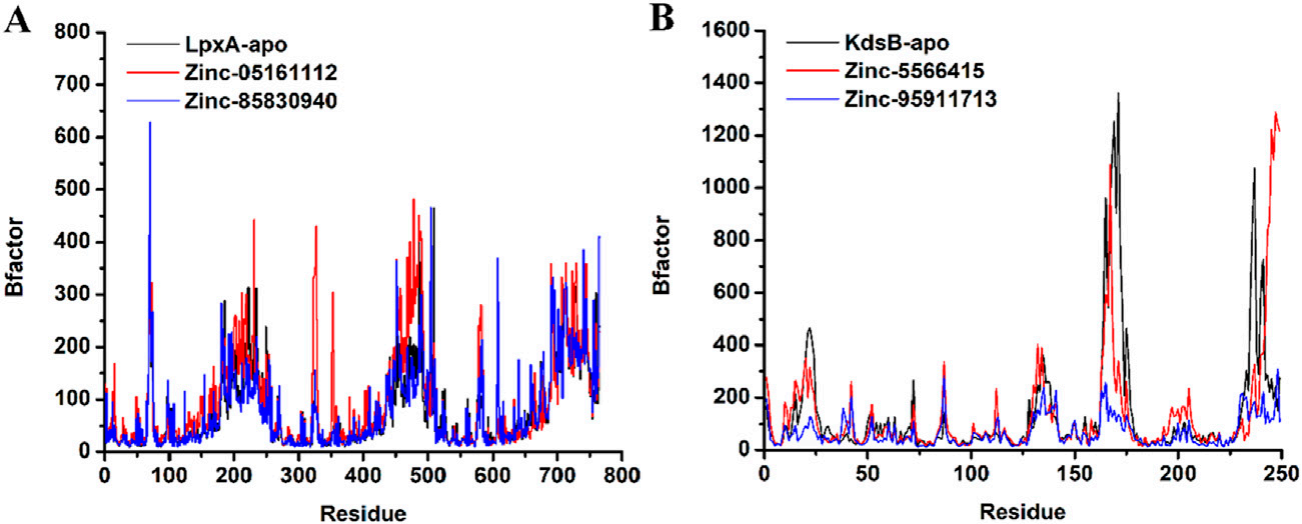
β-Factor plot of the *LpxA* **(A)** and the *KdsB* **(B)** for apo and ligand-bound complex over 100ns simulation run.

β-Factor explains the thermal stability and flexibility of the protein over some time. The quantity of β-factor is measured in RMSF. Its value on the level of localized atomic fluctuation collectively contributes to the global vibrational movement of the protein and its thermal stability. The β-factor value for ZINC05161112 at 327th residue and 352nd residue exhibited higher thermal instability that was exactly in line with the RMSF results. Similarly, for ZINC85830940, residue 607th demonstrated higher thermal instability in that region. Few residues lying between 450th – 460th position for both ZINC compounds also showed a certain degree of thermal instability and thermal flexibility and were cross-checked with the corresponding RMSF observations and were found similar. In contrast, the protein *KdsB-*apo and the ZINC5566415-bound *KdsB* exhibited fewer elevated thermal instabilities at around the 170th residue and at the 235th residue. Interestingly, the ZINC95911713-bound *KdsB* exhibited comparatively global vibrational and thermal stabilities at the aforementioned position as well as at other positions during the whole simulation period. Collectively, the β-factor and the RMSF values are in line, showing the accuracy of the simulation (Figure 14).

#### 3.7.4 Radius of gyration (Rg)

**FIGURE 15:**
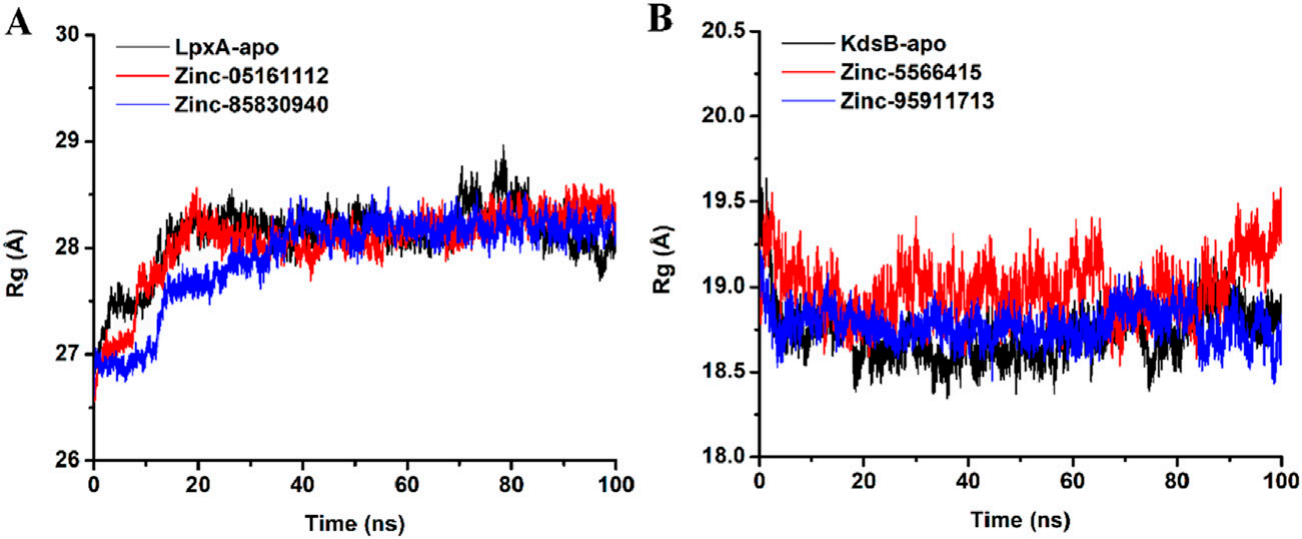
Radius of gyration (Rg) plot of the *LpxA* **(A)** and the *KdsB* **(B)** for apo and ligand-bound complex over 100ns simulation run.

The radius of gyration was calculated to evaluate the structural compactness as a function of time for apo and protein-ligand complex. The average Rg value for apoprotein was recorded as 28.1Å with an SD of 0.3 while ZINC05161112 bound the *LpxA* protein exhibited an average Rg value of 28.05 with an SD of 0.3 and ZINC85830940 exhibited an average Rg score of 27.93 with Sd of 0.4. For the *KdsB* apo protein, the Rg value was recorded as 18.73 with an SD of 0.16, while ZINC5566415 bound *KdsB* exhibited 18.99 with an SD of 0.16 and ZINC95911713 exhibited 18.77 with an SD of 0.1. These values suggest that the overall globularity of the protein upon ligand binding was neither decreased nor increased but remained consistent with the apoprotein structure (Figure 15).

#### 3.7.5 Evaluation of hydrogen bonds

**FIGURE 16:**
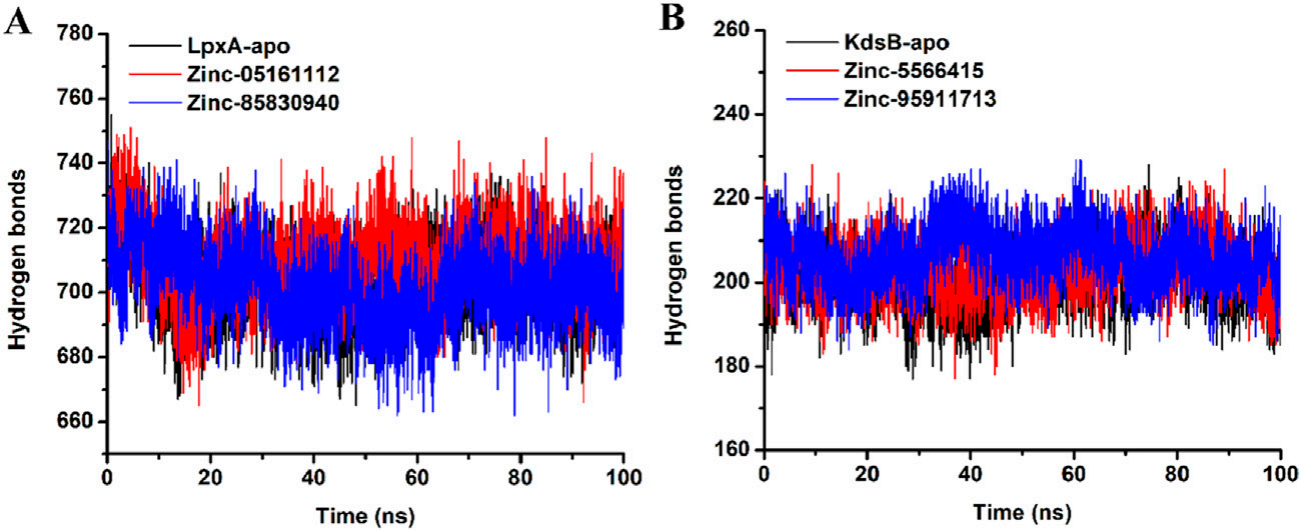
H-bonds plot of the *LpxA* **(A)** and the *KdsB* **(B)** for apo and ligand-bound complex over 100ns simulation run.

Hydrogen bond analysis provides an essential understanding of the intramolecular hydrogen bond network of apo and the ligand-bound *LpxA* and the *KdsB* proteins. Figure 16 provides deep insights into the Hydrogen bond network of both the apoproteins structures and ligand binding. Careful evaluation of the number of H-bonds revealed that the average number of H-bonds for the apo *LpxA* protein was 702.9, while upon binding of ZINC05161112, the average number of H-bonds increased to 709.7, and upon binding of ZINC85830940, the average H-bonds decreased to 701.8. The results depict that upon ligand binding, the number of H-bonds significantly varied between both the *LpxA* proteins. For the *KdsB* protein, the apo form exhibited 200.6 average H-bonds, while the ligand-bound *KdsB* protein recorded the average number of H-bonds as 203.10 and 206.48 for ZINC5566415 and ZINC95911713, respectively (Figure 16B). The H-bond number significantly increased upon ligand binding which depicts that the *KdsB* protein achieved stability and favors the complex form rather than the apo form.

#### 3.7.6 Solvent-Accessible Surface Area

**FIGURE 17:**
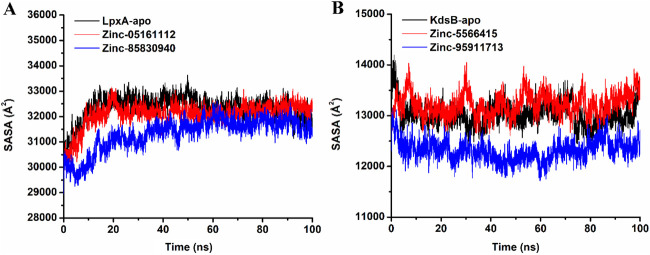
Solvent-accessible surface area (SASA) plot of the *LpxA* **(A)** and the *KdsB* **(B)** for apo and ligand-bound complex over 100ns simulation run.

The SASA (Solvent-accessible surface area) parameter determines how much of the protein surface is accessible to the aqueous solvent. The SASA formula can be used to estimate the magnitude of conformational fluctuations that occurred during contact. The plot of SASA values vs time for all complexes is shown in Figure 17. Through molecular dynamic simulations, the average SASA of apo-*LpxA* is 32,324.7, while for ZINC05161112 bound protein complex was 32,088.23 and ZINC85830940 bound *LpxA* was recorded as 31,323.2. On the other hand, the apo-*KdsB* protein exhibited 13,025, while ZINC5566415 and ZINC95911713 exhibited 13,225.8 and 12,315.7, respectively. All of the ligand-bound bound-*LpxA* complexes exhibited lower SASA values, while for the *KdsB,* the SASA values for ZINC5566415 showed inconsistent increase at different time intervals while having a steep decrease for ZINC95911713. As a result of our SASA research, we have observed that the *LpxA* protein-ligand complex is rather more stable than the *KdsB* complex.

*LpxA*: *LpxA* (UDP-N-acetylglucosamine O-acyltransferase) is essential for lipid A synthesis, a core component of lipopolysaccharide (LPS) in Gram-negative bacteria, including Alistipes. LPS forms a protective outer layer that enables bacterial evasion of immune responses and triggers strong inflammatory responses by activating Toll-like receptor 4 (TLR4) in host cells, a mechanism associated with chronic intestinal inflammation and gut dysbiosis (Raetz and Whitfield, 2002). By inhibiting *LpxA*, lipid A biosynthesis can be disrupted, weakening the bacterial cell membrane and enhancing susceptibility to immune clearance (Hornef et al., 2002). Studies have shown that targeting *LpxA* can significantly reduce bacterial viability and virulence, making it a promising therapeutic target for controlling bacterial infections (Emiola et al., 2015; Wang and Quinn, 2010).

*KdsB*: *KdsB* (3-deoxy-D-manno-octulosonate cytidylyltransferase) is another crucial enzyme in LPS biosynthesis (Schmidt et al., 2011). It facilitates the incorporation of Kdo (3-deoxy-D-manno-octulosonic acid) into lipid A, which is vital for bacterial viability. Inhibiting *KdsB* disrupts LPS integrity, making the bacterial outer membrane more susceptible to immune responses and reducing virulence. Mutations in *KdsB* are lethal for bacteria, indicating its essential role in maintaining LPS structure and function (Alfahemi, 2020). Research supports the development of small-molecule inhibitors for *KdsB*, demonstrating its potential as a drug target for antimicrobial therapies (Schmidt et al., 2011; Ahmad et al., 2019).

It is important to note that in the present work, the identified compounds are from ZINC and TCM libraries, which are commercially available and are ready to “prepare on demand”. Furthermore, the ADMET and pharmacokinetic profiling of the selected identified compounds/inhibitors primarily elucidate their safety measures, which renders them “best hits” for futuristic *in vitro* and other mechanistic analyses. The overall interaction scores, number of H-bonds, and other thermodynamic parameters are good predicted indicators and thus hypothesize the inhibitory effectiveness of all the final hits.

## 4 Conclusion

The increasing resistance of bacterial pathogens to antibiotics underscores the urgent need for novel drug targets and therapeutic strategies. In this study, we employed a subtractive genomics and pangenome analysis approach to identify potential therapeutic targets in *Alistipes*, a gut-associated opportunistic pathogen. Through a systematic computational pipeline, *LpxA* and *KdsB* were identified as essential, non-host homologous, and druggable proteins, making them promising targets for antibacterial drug development. Further molecular docking and ADMET profiling of these targets against two extensive compound libraries, the ZINC database (n = 11,993) and the Traditional Chinese Medicine (TCM) database (n = 36,043), led to the identification of ZINC05161112, ZINC85530940, ZINC05566415, and ZINC9591171 as potential inhibitors. Molecular dynamics (MD) simulations further confirmed the stability and binding efficacy of these compounds within the active sites of *LpxA* and *KdsB*, suggesting their potential as lead drug candidates.

While this study provides a strong computational framework for robust drug discovery that addresses an urgent need for novel antimicrobial strategies, there are certain limitations. The lack of experimental validation remains a key point at this stage, and future studies are required that should focus on *in vitro* and *in vivo* assessments to confirm the biological efficacy of the identified inhibitors. Additionally, structural refinement of the lead compounds may enhance their pharmacokinetic properties and therapeutic potential. Further exploration of *Alistipes* metabolic pathways could also uncover additional druggable targets to complement the inhibition of *LpxA* and *KdsB*.

In conclusion, with a certain degree of modifications, this research and other similar approaches lead directly or indirectly to fulfill the objective of identifying novel drug targets in *Alistipes* and provide a comprehensive computational strategy for structure-based drug discovery (Hassan et al., 2012; 2018; 2023; Pethick et al., 2012b; 2012a; Dorella et al., 2013; Basharat et al., 2022; Afzal et al., 2023; Fatima et al., 2023; Kalhor et al., 2023; Alqurashi et al., 2024). The findings serve as a foundation for future experimental validation and clinical investigations aiming to develop targeted therapeutic interventions against *Alistipes*-associated infections.

## Data Availability

The original contributions presented in the study are included in the article/supplementary material, further inquiries can be directed to the corresponding author.
